# Mitigating the charging rush hour

**DOI:** 10.1016/j.heliyon.2024.e40258

**Published:** 2024-11-12

**Authors:** Milica Savanovic, Lisa Göberndorfer, Georg Jäger

**Affiliations:** Institute of Environmental Systems Sciences, University of Graz, Merangasse 18, Graz, 8010, Austria

**Keywords:** E-mobility, Charging, Electricity demand, Sustainability, Sustainable mobility, Electric cars, Renewable energy

## Abstract

Great effort is put into making our mobility system more sustainable in order to mitigate climate change. One corner stone of this endeavour is the transition from internal combustion engines to electric engines for private cars. This transition, however, introduces new challenges, especially regarding the demand for electrical energy from renewable sources. One emerging phenomenon is the so-called charging rush hour, i.e. a sharp demand spike when many people arrive home and begin charging their electric cars. In this study we use an agent-based model calibrated with empirical data on mobility behaviour to investigate strategies for mitigating this charging rush hour. Studied counter strategies include telecommuting and the possibility to charge the car at work. Our findings show that the baseline peak of 65 MW per 100 000 people can only be reduced to 55 MW per 100 000 people even when combining multiple strategies. Thus the small incentives and policy changes investigated here are not enough to solve the problem of the charging rush hour and more disruptive changes to our mobility system are required.

## Highlights

• Charging a fleet of electric cars will produce a sharp peak in electricity demand in the evening.
• This charging rush hour peak can reach 65 MW per 100 000 people on national level (Austria).
• No investigated policy intervention can reduce it below 40 MW per 100 000 people.

## Graphical abstract

**Figure:**
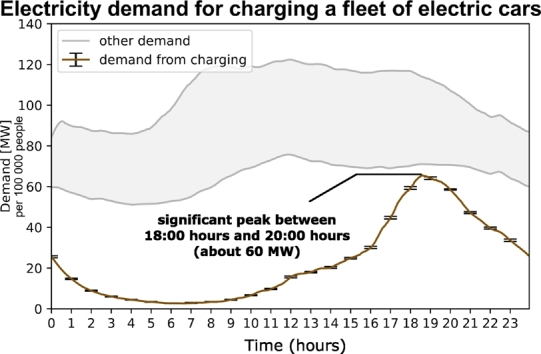

## 1 Introduction

The transport sector is responsible for a large amount of global greenhouse gas (GHG) emissions [1] and shows an increasing trend [2]. Disruptive changes to our mobility system are needed in order to reduce these emissions fast enough and limit global warming to an acceptable level.

The exact nature of these disruptive changes and which policies or regulations are needed to pave the way for a sustainable mobility system is currently debated scientifically as well as politically [3], [4], [5]. There is, however, consensus that a combination of different ideas is required. These are ranging from behavioural changes like a shift to public transport or increased digital interactions over policy and regulation changes that decrease the usage of private cars to technological innovations such as electric cars [6], [7], [8], [4]. Especially e-mobility is heavily investigated as it is seen as a necessary component of a sustainable mobility system [1], [9], [10], [11]. Current research regarding the shift to electric mobility can be categorised into two areas: First, the innovation diffusion perspective in which researchers try to identify an optimal path so that e-mobility can be adopted by the majority of people in the next decades [12], [13], [14], [15]. Secondly, the technological perspective in which the focus is on optimising electric motors, batteries and charging infrastructure [16], [17], [18], [19], [20], [21], [22]. Despite this great research interest, the problems that arise after the transition to fully electric mobility receive less attention.

Nevertheless, there are studies that investigate the electricity demand of a fully electric fleet such as Kapustin and Grushevenko (2020) and Krause et al. (2020) [23], [24]. They are complemented by case studies that focus on single regions or mobility systems [25], [26], [27]. De Gennaro et al. (2014) estimate the additional demand as follows: Domestic energy consumption increases by nearly 20% when replacing 28% of private cars with electric cars in an urban environment [28].

In addition to the rising demand for renewable energy [1], [29], there is also the problem of the so-called charging rush hour, i.e. a peak in electricity demand at times when a large fraction of the population begins charging their electric vehicles. This effect was approximated by Jäger (2019) [30] with the result that this demand peak is significant and requires further investigation. The charging rush hour was further explored quantitatively in Göberndorfer et al. (2023) [31]. In the latter, a case-study region was selected and its mobility extrapolated to the year 2040 when full private e-mobility seems feasible [9], [32], [33]. An agent-based model was designed to calculate the size and the time of occurrence of this charging rush hour. The main result of these simulations was that the resulting peak demand of roughly 800 Watt per person was significant, as it increases the usual peak from domestic and industrial electricity demand by up to 80% [31]. Thus, it is imperative to examine the problem of the charging rush hour more closely to prevent a possible future electricity issue.

Specifically, we want to address the following limitations of previous studies: Jäger (2019) [30] investigates only a single solution to the charging rush hour problem. Furthermore, the times at which the cars are charged are derived from approximating equations and not from empirical data, making the results less realistic. While Göberndorfer et al. (2023) [31] solved the problem of realism by incorporating empirical data related to charging behaviour, that study was limited to a single geographical region. Additionally, it only investigated the magnitude of the problem, but did not analyse any solutions. This identified research gap leads to the following research questions:

• **RQ1:** Does the problem of the charging rush hour occur on a national level or is it restricted to specific regions?
• **RQ2:** Which counter-strategies are effective against the charging rush hour?
• **RQ3:** Is the combination of several counter-strategies more efficient than a single strategy?

The aim of this study is to explore approaches to flatten the demand peak arising from e-mobility that do not rely on smart charging or similar technology with concerns regarding privacy and data protection [34], [35]. Various possible solutions focusing on policy measures are taken into account and their effects on the electricity demand are investigated using an agent-based model. The primary goal is to find strategies to flatten the demand curve, relative to what would be expected without interventions. Reducing the overall electricity demand is only a secondary objective.

By using the agent-based computer model it is possible to investigate strategies from different fields within the same framework. The most promising domains that can be explored are:

• rules and regulations
• technological changes and innovations
• behavioural changes driven by climate responsibility
• behavioural changes driven by convenience or economic reasons

Rules and regulations have the advantage that the change is imminent and every person is required by law to adhere to them. It is, however, sometimes difficult to change regional, national or even global policies regarding electric transport [36], [37], [38].

By contrast, technological change is more often seen as an advancement and generally better accepted by the population. Nevertheless, most technological advancements such as faster charging speeds only increase the problem of the charging rush hour [30], [31].

Behavioural changes that are driven by a sense of climate responsibility are also commonly discussed in literature [39], [40], [41], [42]. Climate responsibility, however, does not necessarily lead to beneficial behaviour in regard to the charging rush hour. The switch to e-mobility itself is perceived as climate-friendly [43]. Meanwhile the positive environmental impact of using or charging your electric car responsibly is not that obvious. Thus, even people with highly pro-environmental behaviour could have little incentive to change their mobility behaviour.

More effective drivers for behavioural change come from economic reasons or simply from convenience. Especially the charging convenience has a high impact on the adoption of private electric mobility [36] and consequently on the mobility behaviour after adoption.

Using these opportunities to facilitate behavioural change, several scenarios of future mobility behaviour are imaginable. In accordance with the main goal of this study we investigate the most promising, accessible and reasonable strategies and quantify their possible effect on the demand curve.

Six strategies were selected for this analysis; a short overview is given in Table 1.

**Table 1:** Overview of investigated strategies.

	Strategy Name	Short Description	Free Parameter
1	Different Charging Rate	Charging is performed with a different charging rate	charging rate [kW]
2	Random Delay	Charging does not start immediately after the car is plugged in since there is a random delay	maximum of the random delay [minutes]
3	Flexible Working Hours	People do not start their commute at a fixed time but within a given period of time	population affected [%]
4	Teleworking	People work from home	population affected [%]
5	Charge at Work	People charge their car at their workplace	population affected [%]
6	Combined	Combination of strategies 1, 4 and 5	same as 1, 4 and 5

In strategy 1 ***Different Charging Rate*** we investigate the effect of a simple idea: By limiting the maximum charging rate to a certain value, cars charge more slowly and the demand is therefore smoothed out over longer periods of time. Downsides of this idea are the rather complicated implementation and possibly lower acceptance among the population since charging time is highly relevant for the convenience of electric car users [44]. In addition, the effect of faster charging rates is investigated.

In strategy 2 ***Random Delay*** the following is considered: To distribute the charging demand of a complete fleet of electric cars over a longer period of time without the need for a smart grid or any communication between cars, a random delay between plugging in the car and the actual start of the charging process could be used [30]. The technical implementation would be simple, since only a timer and a random number generator need to be added to a charging station. As long as it can be guaranteed that the car will be sufficiently charged when the user typically needs it, there are few disadvantages for the user.

Strategy 3 ***Flexible Working Hours*** investigates a currently observed change of our labour market. Today's industry and labour market feature various significant trends [45], among them is an increased need or desire for flexible working hours [46] with many related advantages and disadvantages [47]. One advantage in terms of mobility is that by increasing the flexibility of working hours, commuting times will also be more flexible. People tend to avoid travelling during peak hours, therefore this naturally leads to less congestion and a smaller peak in charging demand.

In strategy 4 ***Teleworking*** we investigate a similar trend. Since the COVID-19 pandemic more people work remotely [48], [49], [50], however, this does not necessarily lead to a linear decrease in mobility. The current opinion in research considering teleworking and path reduction is ambiguous. Ellder et al. (2020) find a path length decrease and mobility mode shift in teleworkers [51], [52]. A review article by Hostettler et al. (2022) points out that current teleworking related studies observing path length reduction [51] (and emissions reduction [53], [54], [55], [56]) are mostly considering just work related paths [57]. Contrary to this, a number of studies that find a positive correlation between path length or number of paths and teleworking consider all possible paths during the day [58], [52], [59], [57]. Family and household related chores can be responsible for that effect as these are often performed by car and, given that the car is available during teleworking, may be performed by other members of the household at different times [52], [60]. While it is still unclear whether teleworking actually decreases car mobility, it is evident that car travel times change and mobility occurs more often outside the commuting peak hours.

In strategy 5 ***Charge at Work*** we explore the possibility of charging the electric car at work. A recent study [61] suggests that workplace-related charging is the second most important charging opportunity after charging at home. According to Hardman et al. (2018) charging at the workplace is currently making up for 15-25% of total charging of commuters [61]. Whilst this number may differ from region to region and between working groups, there is a visible trend towards the importance of available charging stations at the workplace for the consumer [62].

In strategy 6 ***Combined*** we look at synergy effects and other interactions by combining most of the already mentioned strategies. Strategies 3 to 5 can be combined in a straightforward manner while strategies 1 and 2 are somewhat exclusive as they slow down the charging process and shift the charging times, respectively. Therefore we choose strategy 1 *Different Charging Rate* as the more promising one based on preliminary results. We also exclude strategy 3 from this combination. Thus we combine strategies 1 *Different Charging Rate*, 4 *Teleworking*, and 5 *Charge at Work*. The disadvantage of combining such policies is that the overall effect will be lower than the sum of individual effects, e.g. the advantage of being able to charge at work will be lower if more people work from home. The overall advantage of this combination, however, is that individual policies with lower intensity are better accepted by the population.

Each of these strategies has a free parameter, as shown in Table 1. Using this variable, a parameter sweep can be performed and the performance of each policy can be analysed at different intensities. Therefore, this analysis does not require precise knowledge or a forecast of currently unknown values such as the percentage of people working from home in the future. By scanning a large range of possible parameter values, general insights into the demand for electricity for the investigates strategies are obtained.

The used model and details about the implementation of the aforementioned strategies can be found in the section *Methods*. Our findings are presented in Section *Results*. The Section *Discussion* closes with a summary of the results, as well as limitations of this study and an outlook.

## 2 Methods

The agent-based model used for this study was developed in Göberndorfer et al. (2023) [31]. The model description presented here will closely follow that publication, in which additional information regarding data and the originally investigated region can be found [31].

The goal of the model is to calculate the electricity demand peak originating from charging private electric cars. In order to generate agents with realistic mobility behaviour, we need detailed information about the trips people take with their private cars, including:

• number of trips per day
• length of each trip
• start time of each trip
• end time of each trip
• reason for each trip (commute, shopping, etc.)

The goal of Göberndorfer et al. [31] was to find the resulting electricity demand in a specific region in 2040, a time when fully electric private mobility seems feasible [9], [32], [33], hence data from this region was used. The main data source was a survey [60] in which roughly 38 000 people in Austria participated by giving detailed reports on their mobility behaviour. This survey provides detailed information about all trips that participants performed during a randomly chosen day. Most importantly, they reported trip length, duration, time, reason and their chosen mode of transport. This information is sufficient to generate agent archetypes, i.e. typical mobility patterns that the model can randomly draw from. For details on this process, we refer to [31].

The current study, however, is not limited to a specific regional scope, so we use data from the full data set. Although mobility behaviour is very dependent on local conditions, they mainly affect the actual size and time of the demand peak. The effectiveness of a strategy is, on the contrary, not that sensitive to specific regional characteristics. Therefore, even though we use a mobility survey from a specific country [60] as model input, our findings can be generalised to other regions. Limitations of this generalisation are presented in the Section *Discussion*.

The model itself is composed of three steps:

• Initialisation
• Driving and Charging
• Aggregation

In the initialisation step the goal is to generate all the agents required for a simulation run. For this either a fixed number of agents or a fixed amount of driven kilometres can be chosen. Additionally, a region type (urban, suburban, rural or mixed) can be selected. Based on these parameters, a population of agents is generated by randomly drawing information from a full day of mobility behaviour from the data set. To avoid numerical artefacts that would arise, we randomise all trip times by up to ± 15 minutes. This randomisation preserves the overall structure of mobility while preventing artificial cluster points. Other data like trip reason and length is taken directly from the data set. The initialisation step is finished once the desired number of agents has been generated.

The main step of the simulation relates to driving and charging. Each agent performs all the trips that are given in its behavioural data. After the last trip of the day the agents start charging their electric cars. The underlying assumption is that people charge their cars every day and start the charging process as soon as they arrive home. While this is the most common behaviour [63], other charging strategies or habits are possible. However, single exceptions do not influence the overall results significantly. Even if people only charge three times a week [64], the overall electricity demand will be very similar on average since anomalies even out in a big enough system.

The time at which each agent charges its car can best be described by the starting time ($C_{start}$) and the end time ($C_{end}$). While $C_{start}$ can be directly taken from the mobility behaviour as the end time of the last trip of the day, $C_{end}$ needs to be calculated:

(1) $$C_{end}=C_{start}+C_{dur}$$

The duration of the charging process $C_{dur}$ depends on the distance driven that day (*d*), the consumption rate of the vehicle (*cor*), the charging loss (*chl*), and the charging rate (*chr*):

(2) $$C_{dur}=d⁎cor⁎(1+chl)\;/\;chr$$

The distance *d* comes directly from the behavioural data of the agent. For the consumption rate of the vehicle we use an average value of 0.216 kWh/km, a value given by the Environment Agency Austria in 2023 [65]. As a default value for the charging rate we chose 11 kW, since this is the most realistic charging speed for charging at home in the future [66], [67], [68]. For the charging loss we use 0.15 (representing a charging loss of 15%), which is in line with literature and experimental studies [69], [70].

Once every agent performed all trips and we know the start time and the duration of each charging process, the last step in the simulation begins. The electricity demand of each agent is summed up using a time resolution of minutes. This yields the time and the size of the charging rush hour peak.

In order to investigate possible solutions and mitigation strategies for the charging rush hour problem, this model needs to be expanded and adapted for each investigated scenario. This involves implementing a free parameter.

In strategy 1 ***Different Charging Rate*** the agents will not charge their cars with the default value of 11 kW. The charging rate can be chosen as a free parameter.

In strategy 2 ***Random Delay*** the agents will not start the charging process immediately after arriving home. Instead there is a random delay between their arrival time and the start of the charging process. This delay is randomly distributed between 0 and the maximum delay $D_{max}$, which is the free parameter for this scenario.

In strategy 3 ***Flexible Working Hours*** a certain percentage of the agents is subject to flexible working hours. This percentage serves as the free parameter of this scenario. An agent with flexible working hours does not start and end their commute at the times given by their behavioural data but either about two hours before or about two hours after the indicated time (120±15 minutes). While the model is unaffected by changes to the time of the commute to work, it is sensitive to the time of the commute home since this can be responsible for the start of the charging process, if and only if the commute home is the last trip of the day.

In strategy 4 ***Teleworking*** we make the assumption based on the literature discussed in the introduction [57] that car usage does not necessarily decrease when telecommuting, but is shifted to different times of the day. Thus, we remove the commute of teleworking agents and replace it with leisure trips of equal length. For leisure trips we make the assumption that they are randomly distributed around noon with a standard deviation of two hours. This is a reasonable assumption since trips that are only possible as a consequence of teleworking should occur during usual business hours. Again, the free parameter of this strategy is the percentage of affected agents.

Strategy 5 ***Charge at Work*** requires more sophisticated expansions than previous strategies. We need to remove the assumption that agents charge after their last trip of the day. Instead, some agents have the ability to charge at the end of their commute to work. However, since we have detailed information about each trip, it is possible to find these trips and start the charging process at the correct time. Note, that the duration of the charging process remains the same since the distance driven in the last 24 hours does not depend on the start time of the charging process. The free parameter of this strategy is the percentage of agents that can charge at work.

Strategy 6 ***Combined*** is a combination of all expansions introduced for strategies 2, 4 and 5. Consequently there are three free parameters, that can be chosen independently.

With these enhanced models it is now possible to investigate the effects of the suggested strategies on the charging rush hour. Results of this investigation are shown in the following section.

## 3 Results

We performed simulations with the model detailed above to calculate electricity demand curves. Every variant of every strategy was simulated ten times and the mean values were calculated. The results are presented in a consistent way: We depict the electricity demand caused by charging electric cars against the time of the day measured in hours after midnight, i.e. t=13 corresponds to 13:00 hours. The magnitude of change in the strategy variants is shown in different shades, starting from brown (low intensity) over yellow (medium intensity) to green (high intensity).

**Figure 1:**
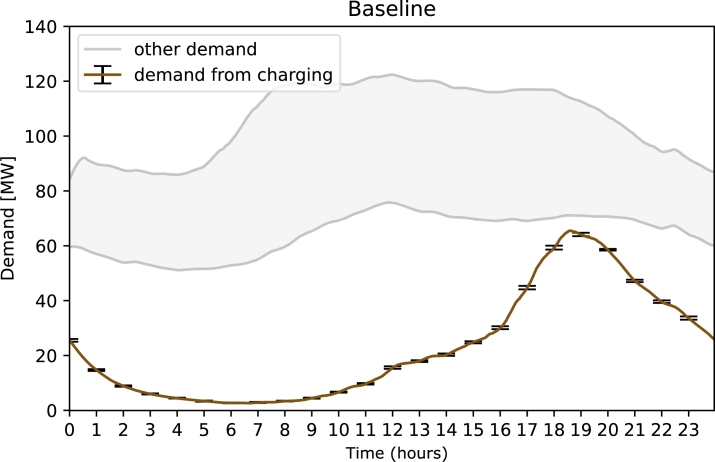
Baseline electricity demand from 100 000 people when all cars are powered by electric engines. The black error bars show the standard deviation of the ten averaged simulations. For comparison, the grey area shows realistic ranges for the electricity demand (domestic and industrial) of a region with 100 000 inhabitants excluding EV charging. The x-axis uses 24h format.

As a first baseline, Fig. 1 shows the results of a scenario without any intervention, i.e. the demand peak that results from e-mobility in a mobility system similar to the one we have today with the exception that all cars are powered by electric engines. The population is set to 100 000 people with a realistic distribution of age, urbanity and mobility behaviour [60]. This leads to a sharp peak in electricity demand of 60 MW per 100 000 people at around 19:00 hours. This is a significant peak, as it is comparable to the overall electricity demand of the country (roughly 60-100 MW). This means, peak electricity demand could double on a national level, which has severe implications on the grid: Even today, with no fully electric fleet, the Austrian power grid relied on redispatch or grid reserve capacity on up to 300 days a year, leading to high costs. [71] The demand spike from charging a fully electric fleet would aggravate this crisis. Thus, it is imperative to investigate the effect of counter strategies, which we will do in the following.

**Figure 2:**
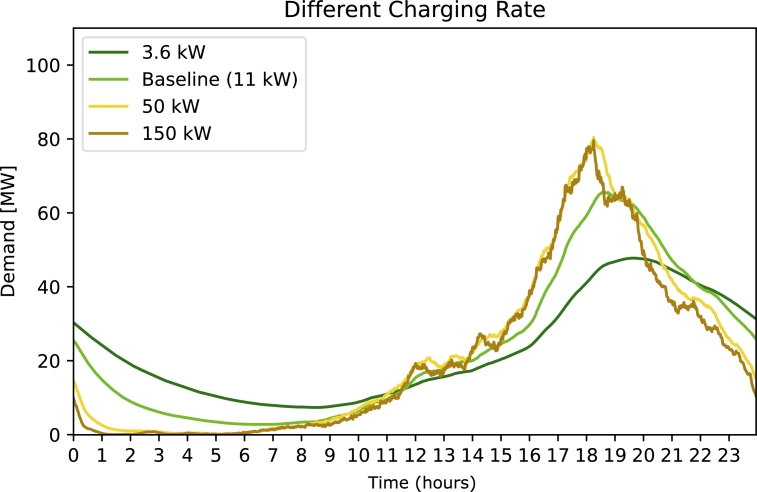
Electricity demand for strategy 1 Different Charging Rate. The free parameter (legend) is the used charging rate.

In Fig. 2 we see the resulting demand of the strategy ***Different Charging Rate***. Limiting the charging rate constitutes a significant loss of comfort, however the strategy is quite effective and limiting the charging rate to 3.6 kW reduces the peak to roughly 45 MW. Note, that the demand does not drop to 0 in the morning hours, which suggests that not all cars are fully charged in the morning. Reasons and consequences of this are specified in the Section *Discussion*. For faster charging rates, the demand peak increases to roughly 80 MW for both 50 kW and 150 kW, which is in line with an analysis performed in [31].

**Figure 3:**
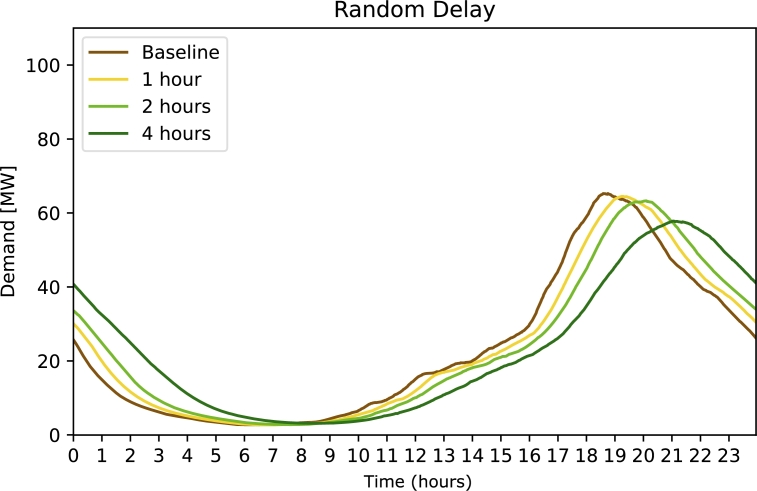
Electricity demand for strategy 2 Randomly Delayed Charging. The free parameter (legend) is the maximally used delay.

Fig. 3 shows the effect of the strategy ***Random Delay***. For a low value of the maximum allowed delay (1 hour) there is no significant decrease in peak demand. Larger delays (4 hours) reduce the peak slightly.

**Figure 4:**
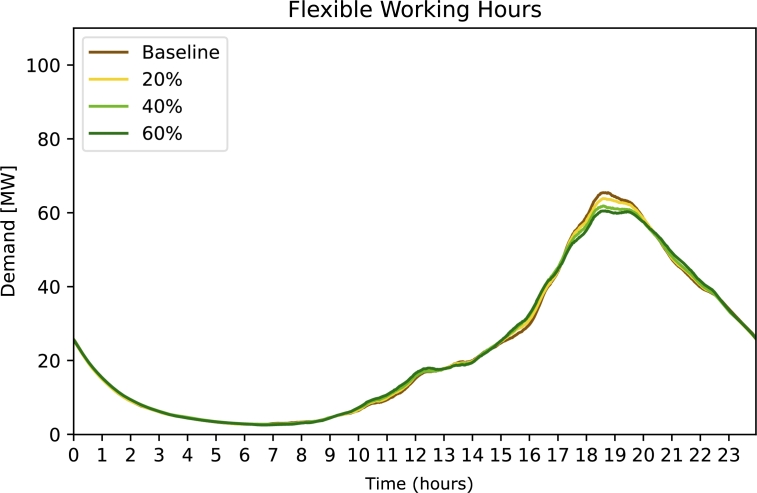
Electricity demand for strategy 3 Flexible Working Hours. The free parameter (legend) is the percentage of affected population.

Fig. 4 depicts the effect of the strategy ***Flexible Working Hours***. Even with a high intensity (60% of people affected) the demand peak is nearly as high as in the baseline.

**Figure 5:**
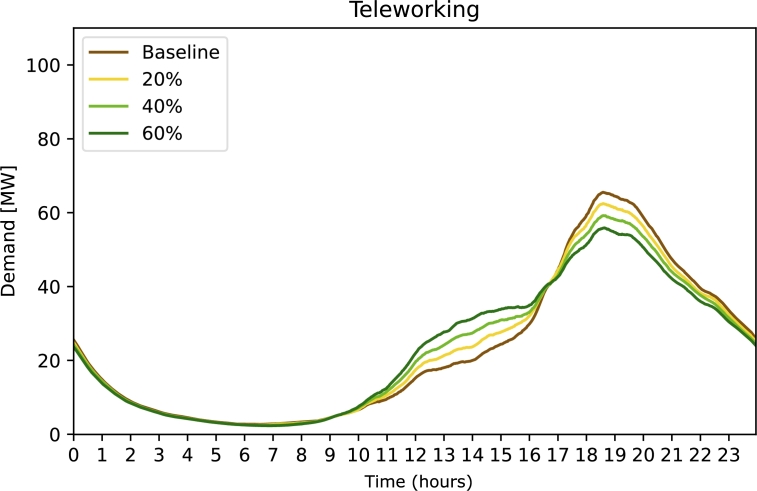
Electricity demand for strategy 4 Teleworking. The free parameter (legend) is the percentage of affected population.

Fig. 5 shows the electricity demand in strategy ***Teleworking***. We see a moderate flattening of the peak at 19:00 hours accompanied by increased electricity demand at noon.

**Figure 6:**
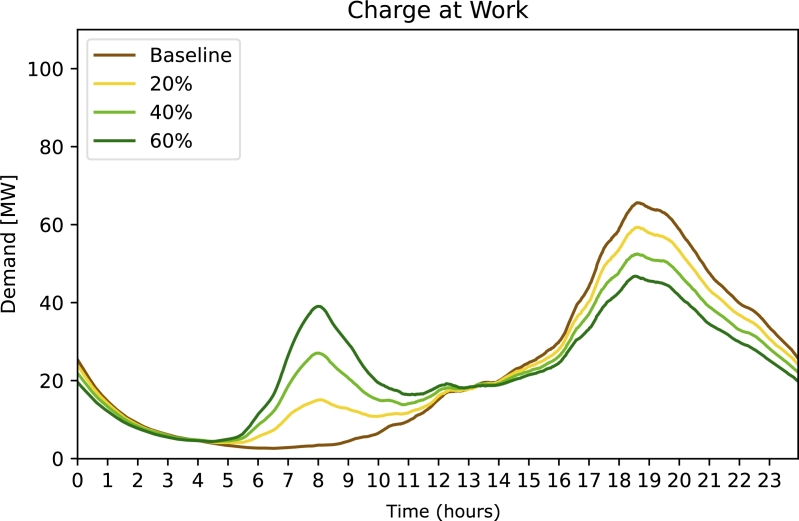
Electricity demand for strategy 5 Charge at Work. The free parameter (legend) is the percentage of affected population.

In Fig. 6 we notice a shift in demand for strategy ***Charge at Home***. At the time people arrive at work (between 7:00 hours and 8:00 hours) there is a second significant peak. In turn, the electricity demand during the charging rush hour is decreased. This leads to an electricity demand peak slightly above 40 MW.

**Figure 7:**
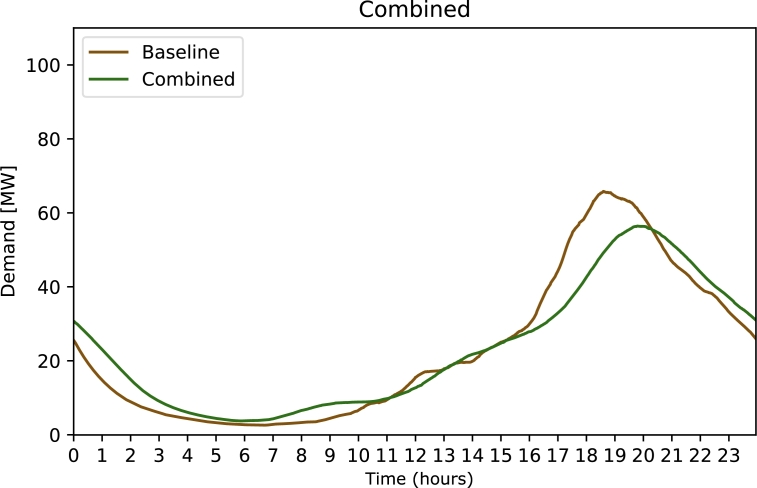
Electricity demand for strategy 6 Combined, combining strategies 2, 4 and 5.

Fig. 7 shows the results of strategy ***Combined***, a combination of a average charging rate of 8.78 kW (corresponding to 70% of the population charging with 11 kW and 30% of the population charging with 3.6 kW), teleworking for 25% of the population and the ability to charge at work for 10% of the population. We see that this combination is a viable way to reduce the charging rush hour demand peak to about 55 MW per 100 000 people.

## 4 Discussion

We investigated the ability of various strategies to reduce the peak electricity demand caused by the charging rush hour. Our findings suggest that no investigated strategy is able to reduce this demand peak down to 50%. The strategies Charge at Work as well as the combined strategy lead to a peak decrease of about 15%. Hence we conclude that no simple policy intervention is sufficient to reduce the electricity demand caused by the charging rush hour to an acceptable level. More fundamental changes to our mobility system and an expansion of the electrical grid, for example by introducing a smart grid, are necessary for a sustainable solution.

When taking a closer look at the strategy results and their interpretations, we can derive the reasons for their performance.

Strategy 1 ***Different Charging Rate*** reduces the demand peak significantly, when charging rate is limited to 3.6 kW. The downside is that not all cars can finish their charging process over night. Although people are usually not that concerned about the charging time when charging at home [63], [72], most of them still need their car to be fully charged in the morning. Thus, lowering the charging rate will not be easily acceptable. Furthermore, it is expected that by increasing the amount of electric vehicles the charging rate must also rise, specifically when charging in public locations [73]. Since higher charging speeds only increase the size of the demand peak, we can conclude, that neither lowering the charging rate to 3.6 kW, nor increasing it, is a promising candidate for mitigating the charging rush hour.

Strategy 2 ***Random Delay*** shows only minimal improvements, but its technical implementation would be simple. The demand peak is not only flattened but also moved to a later time of the day in which the usual domestic electricity demand is lower. This comes without any side effects since the behaviour of the users is not affected.

Strategy 3 ***Flexible Working Hours*** shows only a insignificant effect on the peak electricity demand even when 60% of the population are affected. This has different reasons: Depending on the distance driven that day, a charging process can take more than two hours. In that case, starting the process up to two hours earlier has no effect on the peak since the car is still charging during the charging rush hour. A second reason is the fact that the commute home is not the last drive of the day for many people and hence does not change the start time of the charging process. In summary, while flexible working hours might have different benefits [74], [75], [76], they are not effective in reducing the load on the electric grid during the charging rush hour.

Strategy 4 ***Teleworking*** is more effective in reducing the electricity demand peak than strategy 3, yet the effect is still limited. This strategy is also most affected by assumptions (see Section *Introduction* for details); first and foremost the assumption that teleworking does not reduce the demand for mobility significantly but rather shifts it to different times of day. Without this assumption results will be different and peak demand will be lower. Nevertheless, the effect of teleworking on mobility is still a matter of current research and we expect that with teleworking on the rise in many countries [77], new insights will be gained in the next years. For now, under the pessimistic assumption that the sum of the driven kilometres remains constant with teleworking, it is not a suitable strategy to combat the charging rush hour.

Strategy 5 ***Charge at Work*** shows the biggest improvement of all considered strategies. However, the investigated intensity of 60% of all workers being able to charge their car at work requires a significant investment in infrastructure. We can also see a peak in the morning hours when people arrive at work. In this simulation this peak is rather sharp due to the pessimistic assumption that everybody charges their car as soon as they arrive at work. In reality, not everybody will be able to charge right away and this peak would smooth out. The main peak at 18:00 hours, however, would not be affected by a more realistic charging behaviour at work. In conclusion, charging at work has a huge potential to reduce the charging rush hour, yet its implementation requires significant infrastructure to be built.

Strategy 6 ***Combined*** uses a combination of strategy parameters to achieve a peak reduction of about 15% while still being viable to be implemented or accepted by the public. 30% of the population using the slow charging rate of 3.6 kW is realistic, under the assumption that all cars need to be nearly fully charged in the morning. Teleworking for 25% of the people seems ambitious but may be viable in the future. Finally, the possibility to charge at work for 10% of workers can be realised with somewhat reasonable investments in infrastructure. All these strategies combined lead to a decrease in peak demand of about 15%.

### 4.1 Limitations

While the developed model is suitable to investigate the effectiveness of certain strategies to mitigate the charging rush hour, there are some limitations. The model includes many simplifications and assumptions and the obtained results might change if these were removed or modified.

One of the biggest assumptions is that people charge their car daily after the last trip of the day. While this is a common behaviour, there are, of course, exceptions. Even if people plan to charge after their last trip, there may be situations in which they are unable to so. If, for example, the last trip of the day was not planned, charging would occur earlier. Aggregated over the whole system, however, we do not expect a significant change in the size of the demand peak and even less influence on the relative effect of each strategy. A different assumption would be possible if one were to investigate a more pessimistic scenario, e.g. everybody charges their cars after their commute home. This assumption will lead to a bigger, more pronounced peak, yet it might not be a good indicator for a realistic situation. Nevertheless, if one is interested in the maximum demand that could occur, it is a useful alternative expansion that could be explored in further research.

A second simplification is that we assume that driving behaviour does not change drastically when using an electric car compared to a car with a combustion engine. In reality, especially if only electric cars are available on the market, mobility behaviour will change. It is currently unclear how this change will look like since several effects interact: The larger investment of buying an electric car would suggest that car availability and thus car usage will decrease [78]. On the other hand, the lower cost per driven kilometre and similar rebound effects might lead to increased car usage [79], [80]. In order to make mobility more sustainable, incentives for public transport, car sharing and smart cities will also have a significant effect on the mobility behaviour of the future. [81].

A further simplification is that we use today's electricity demand of electric cars, although this value might change in the future. This is of special importance to the model: all results are linearly dependent on this parameter, i.e. a 10% higher consumption rate leads to a 10% higher energy demand. Making a prediction or forecast is challenging given that two contrary effects are present. On the one hand, technological improvements make engines, batteries and charging stations more efficient [82], [83]. On the other hand there is a trend towards bigger electric cars with a larger consumption rate [84]. It is unclear how the average consumption rate of a fully electric fleet will look like, hence it is a matter of future research.

### 4.2 Geographical context

While the model and its results can in principle be generalised, there is a certain effect of the chosen data set used to calibrate mobility behaviour on the model. The data set is from Austria, therefore results can be generalised for similar European countries. If the average distance driven by car is much higher or much lower than the roughly 30 km per day used here, it might be necessary to adapt the model. Moreover, if car availability differs, this should be included in the model. Focusing just on the averages is not sufficient here: Consider two scenarios with 1 000 people: In one we have 100 cars, each driving 100 kilometres and in the other we have 1 000 cars, each driving 10 kilometres. Even though the electricity demand is the same and the average distance driven per person is identical, the demand peak of the second scenario is ten times higher. The ratio people vs. cars is highly important and might differ considerably from country to country. The model would also need to be adapted to correctly reflect cultural differences related to working, commuting and working hours. The primary findings on the effectiveness of each strategy to mitigate the charging rush hour can thus be generalised while detailed results about the size and the time of the charging rush hour for specific regions would require mobility behaviour data from the investigated region.

### 4.3 Further research

The model we designed was able to estimate the effect of certain strategies on the demand profile generated by charging electric cars. None of the proposed policies, however, were able to reduce the demand peak drastically enough to solve the problem of the charging rush hour. Alternative ideas for solutions exist, but since they are out of scope for the model presented here, they cannot be investigated in this study. They are, however, important avenues of future research, using different models or even fundamentally different methods. Interesting solutions include:

**Dynamic electricity pricing:** One way to shift electricity demand effectively is dynamic electricity pricing [85], i.e. the concept that the price for electricity varies over time. In its simplest form, electricity could be cheaper at night and more expensive during the day, but more complicated price structures are possible, up to incorporating live data. Especially for charging a car, which is an activity that is often easy to shift in time, compared to more restrictive activities like for example preparing a meal, dynamic prices can lead to a big shift in the demand profile, possibly reducing the demand peaks identified by our study [86]. The demand profiles we generated could even serve as the basis for designing effective price structures and investigating their effects in future research.

**Intelligent charging systems:** A different approach, relying on technology rather than policy, are intelligent charging systems [87]. In these systems, not all cars are charged simultaneously, but some form of intelligent decision process guides the charging with the goal of optimally shaping the demand curve while still adhering to some given boundary conditions. While this solution is very appealing, it would be a huge investment in infrastructure and it is not clear if the acceptance of the population of this technology is large enough to make widespread application possible [88].

**Vehicle to grid solutions:** Improving on the idea of intelligent charging systems, one could introduce vehicle to grid (V2G) [89], [90]. Also this solution relies on a smart grid, i.e. communication between electricity producers and electricity consumers. In addition to all the benefits that an intelligent charging system brings, V2G also enables that surplus electricity (e.g. produced on sunny days around noon) can be stored in the battery of the vehicles and fed back into the grid if needed. Since those batteries have high capacity and can charge and discharge quickly, they can have a big impact on the resulting demand profile [91]. It faces similar challenges with acceptance as other smart grid solutions, but is also limited by the fact that most cars are not available for V2G during business hours, reducing its potential.

**Carsharing:** One main reason for the charging rush hour is societies reliance on private cars. Reducing the amount of cars would also reduce the maximal possible load on the grid, so even with the same traffic volume, carsharing will reduce the electricity demand peak. While carsharing has many other benefits as well, it is limited by low acceptance of the population [92]. Owning a private car is not only more convenient, but can also be a status symbol or serve other purposes [93].

**Mobility as a service:** Expanding on the idea of car sharing, mobility as a service (MaaS) [94] could reduce the demand peak in a similar way. In MaaS concepts, people do not pay for their own means of transport, but for the service of being transported. That way, the actual routing can be performed by a central entity, that has enough data and resources to plan efficiently. Multi-modal travel is also possible, making the share of car usage even smaller.

Investigating those possible solutions and their effect on the electricity demand caused by charging electric car could be a next step to solve the problem of the charging rush hour and, more generally, get a step closer to the overall goal of sustainable mobility.

## 5 Conclusion and policy implications

We used an agent-based model to gain insight into the electricity demand and especially the demand peaks that can occur when charging a fleet of electric cars. By using this method we can now answer the posed research questions:

• **RQ1:** Does the problem of the charging rush hour occur on a national level or is it restricted to specific regions?
  On a national level we identified a sharp demand peak at roughly 19:00 hours. Its size (60 MW / 100 000 people) is comparable to the sum of all other domestic and industrial demand and may therefore be critical for the electrical grid. The charging rush hour is therefore not restricted to specific regions.
• **RQ2:** Which counter-strategies against the charging rush hour are effective?
  By investigating strategies to mitigate this charging rush hour we found that the possibility to charge at work for 40% of the population or a mix of different strategies can flatten the peak by about 15%. However, none of the investigated strategies were able reduce the demand peak by more than a third.
• **RQ3:** Is the combination of several counter-strategies more efficient than a single strategy?
  We did not identify positive synergy effects. Combining different strategies of lower intensity could, however, lead to better acceptance compared to a single strategy of high intensity.

In conclusion, no simple policy intervention is capable of solving the problem of the charging rush hour given today's mobility behaviour, which strongly relies on the private use of cars. In order to achieve a sustainable solution for mobility, more drastic changes to our mobility and energy system are necessary and further research is required.

## CRediT authorship contribution statement

**Milica Savanovic:** Writing – review & editing, Writing – original draft, Visualization, Validation, Software, Methodology, Investigation, Formal analysis, Data curation, Conceptualization. **Lisa Göberndorfer:** Writing – review & editing, Writing – original draft, Visualization, Validation, Software, Methodology, Investigation, Formal analysis, Data curation, Conceptualization. **Georg Jäger:** Writing – review & editing, Writing – original draft, Visualization, Validation, Supervision, Software, Project administration, Methodology, Investigation, Funding acquisition, Formal analysis, Data curation, Conceptualization.

## Declaration of Competing Interest

The authors declare the following financial interests/personal relationships which may be considered as potential competing interests: Milica Savanovic reports financial support was provided by Provincial Government of Styria. Lisa Goeberndorfer reports was provided by Provincial Government of Styria. If there are other authors, they declare that they have no known competing financial interests or personal relationships that could have appeared to influence the work reported in this paper.

## Data Availability

Input data regarding mobility behaviour was obtained from [60] and can be requested for scientific work from the Federal Ministry for Climate Action, Environment, Energy, Mobility, Innovation and Technology, Austria. Output data from Figure 1, Figure 2, Figure 3, Figure 4, Figure 5, Figure 6, Figure 7 can be requested from the authors georg.jaeger@uni-graz.at.
